# Transverse Vibration of Viscoelastic Sandwich Structures: Finite Element Modeling and Experimental Study

**DOI:** 10.3390/ma14247751

**Published:** 2021-12-15

**Authors:** Zhicheng Huang, Jinbo Pan, Ziheng Yang, Xingguo Wang, Fulei Chu

**Affiliations:** 1 School of Mechanical and Electronic Engineering, Jingdezhen Ceramic University, Jingdezhen 333001, China; huangzhicheng@jci.edu.cn (Z.H.); 1920031022@stu.jci.edu.cn (J.P.); 118040400111@stu.jci.edu.cn (Z.Y.); 2 Department of Mechanical Engineering, Tsinghua University, Beijing 100084, China; chufl@mail.tsinghua.edu.cn

**Keywords:** compression damping, transverse vibration, viscoelastic sandwich beam, finite element method

## Abstract

In the present work, the nonlinear vibration behavior of elastic-viscoelastic-elastic sandwich (EVES) beams is studied. A finite element (FE) equation taking intoaccount the transverse compression deformation of the viscoelastic core for the EVES beams is derived. In order toaccurately characterize the frequency-dependent feature of the viscoelastic materials layer, athird-order seven-parameter Biot model isused. A 2-node 8-DOF element is established to discretize the EVES beams. The experimental testing onEVES beams validates the numerical predication of the FE model. Numerical and analytical investigations are carried on a series of EVES beams with different thicknesses. The results indicate that the presented FE model has better accuracy in predicting the natural frequency of the sandwich beams, and in predicting damping, the accuracy is related to the thickness of each layer. The results of this paper have important reference values for the design and optimization of the viscoelastic sandwich structure.

## 1. Introduction

Sandwich structure is a special type of composite structure. It has the ability to significantly reduce weight while maintaining mechanical properties. That is, it has typical characteristics of light weight, high rigidity, and high strength [1].The weight reduction in the sandwich structure brings many benefits, including increased stroke, greater load, and reduced fuel consumption. All of these have a positive impact on costs and reduce the impact on the environment. In addition to the high stiffness-to-weight ratio, sandwich composites can also achieve different functions by choosing different core materials. For example, the use of foam [2,3,4], functionally graded materials [5,6,7], and viscoelastic materials [8,9,10] for the core material can achieve the effects of flame retardant, heat insulation, vibration, and noise reduction. Therefore, sandwich composite structures are more and more widely used in aerospace, automotive, marine, naval industries, and civil engineering.

**Figure 1:**
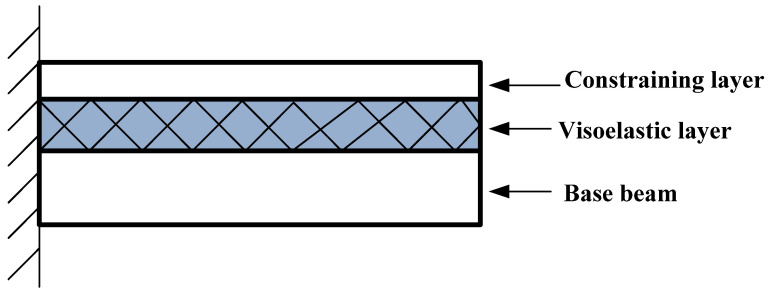
Elastic-viscoelastic-elastic sandwich beam structure.

Figure 1 shows a typical elastic-viscoelastic-elastic sandwich (EVES) beam structure. When the base beam vibrates, the viscoelastic layer will produce the corresponding deformation to dissipate vibration energy. However, what deformation of the viscoelastic layer dissipates energy is a question. There are two main assumptions for the energy dissipation mode of the viscoelastic layer: shear and compression energy dissipation assumptions. When the relative movement of the constraining layer and the base beam layer is parallel to the neutral plane of the sandwich beam, the viscoelastic core layer will produce shear deformation to dissipate vibration energy. When the relative movement of the constrained layer and the base beam layer is perpendicular to the neutral plane of the beam, the viscoelastic core layer will produce compression-tension deformation to dissipate vibration energy. Simply put, the former considers the viscoelastic layer to be incompressible, and the latter considers the viscoelastic layer to be compressible. The former dissipates vibration energy through shear deformation, and the latter dissipates vibration energy through compression deformation.

Most studies adopt the shear energy dissipation assumption. They believe that the transverse displacement of all points on the same cross-section of EVES structure is the same, so there is no compression/tensile deformation in the viscoelastic layer. The relevant research work can be traced back to the 1950s. Ross Kerwin Ungar’s (RKU) model is considered the first analytical model of EVES structure [11,12,13,14]. Later, Ditaranto extended Kerwin’s work and proposed a sixth-order linear homogeneous differential equation to study the free vibration of sandwich beams. However, the calculation results of the model show that the structural loss factor does not depend on the boundary conditions, which is obviously not in line with reality [15]. Mead and Markus modified Ditaranto’s model and proposed the famous Mead-Markus model, which can be applied to various boundary conditions [16,17]. The above-mentioned RKU model and Mead-Markus model were based on the shear assumption in the processing of the displacement domains of the three different layers of the EVEC beam structure. Later, many scholars such as Johnson et al. [18], Galucio et al. [19], Kumar et al. [20], Bilasse et al. [21], Kpeky et al. [22], Hamdaoui et al. [23], and Huang et al. [24,25] developed the FE method for viscoelastic sandwich structure, which were still based on the assumption of shear energy dissipation. Recently, some scholars have also carried out related researches on viscoelastic sandwich structures based on the shear hypothesis. Karmi et al. [26] carried out a dynamic analysis of sandwich beams with a viscoelastic core subjected to a moving load, and the mathematical formulation was based on shear deformation theory. Mario et al. [27] studied the fractional viscoelastic characterization of sandwich beams under time-varying loading. The fractional viscoelasticity is incorporated in the analytical model of a three-layer laminated beam. Maleki-Bigdeli et al. [28] presented an analytical model for the bending problem of a viscoelastic sandwich plate. The generalized Maxwell model was used to describe the viscoelastic response. Amanieh et al. [29] studied the nonlinear vibrationof a sandwich plate. The four-parameter fractional derivative model was used to determine the frequencydependence of the viscoelastic core. Garbowski et al. [30,31] studied the transverse shear stiffness in sandwich plates.

The above-referred papers deal with the analysis of the sandwich structures under theshear energy dissipation assumption. On the other hand, some scholars have found the compression damping of EVES structure. In the 1970s and 1980s, Douglas and Yang [32,33] proved the existence of compression damping through experiments on EVES beams. In addition, based on the assumption of compression energy dissipation, an analytical model (Douglas-Yang model) of the EVES beam was established, ignoring the shear deformation of the viscoelastic layer. They believed that compression damping is the main form in a narrow frequency band centered on the compression resonance frequency of the viscoelastic layer. However, their work did not attract people’s attention at the time. Later, Sisemore et al. [34,35] conducted a more in-depth study on the cantilever EVES beam structure. They not only proved the existence of compression damping experimentally but also established an analytical model of the EVES beam structure based on the assumption of compression energy dissipation. The Sisemore model assumed that the viscoelastic layer was a compressible spring, and the vibration energy was dissipated through the transverse compression of the spring, and there was no shear effect. They proved that the analytical model can predict the resonance frequency of the structure well, but it cannot predict the loss factor well Funari et al. [36] presented a nonlinear approach to investigate the behavior of composite sandwich structures. The core was considered compressible.

It can be seen from the above literature that the research based on the shear energy dissipation assumption of the viscoelasticis relatively in-depth. However, the research based on the assumption of compression energy dissipation is still in its infancy. There are only a few analytical models, and FE models are rarely seen. In engineering practice, EVES structures that need to be designed or analyzed often have complex geometric shapes and boundary conditions and bear complex loads. The analytical method cannot effectively solve these problems. The FE method has shown its excellent applicability and has become the most important method for studying the vibration problems of EVES structures. The study of the FE compression model of the EVES structures has great engineering significance.

In order to make up for this deficiency, in this work, a FE model for EVES beam is established based on the compression energy dissipation assumption. A sandwich composite element with two-node eight degrees of freedom is established. In the process of modeling, it is considered that the energy dissipation is caused by the compression deformation of the viscoelastic layer, and the transverse displacement of the viscoelastic layer is a linear interpolation between the transverse displacement of the constraining layer and base beam layer. The Biot model is used to characterize the frequency-dependent characteristics of viscoelastic materials. Finally, the FE model is verified by comparing with the analytical solutions and experimental values, and some useful conclusions are obtained.

## 2. FE Model for an EVES Beam

### 2.1. Fundamental Assumptions

The present analysis is based on the following assumptions:

1. The structural damping is only caused by the transverse compression/tensile deformation of the viscoelastic sandwich layer;
2. The constraint layer and the base beam are regarded as Euler-Bernoulli beams;
3. Considering the compression deformation of the viscoelastic layer perpendicular to the neutral plane of the beam, it is considered that the base beam layer, the damping layer, and the constraint layer have different deflection functions, and the transverse displacement of the viscoelastic layer is the linear interpolation of the two surface layers;
4. The materials of each layer are firmly pasted, and there is no relative sliding between the layers;
5. The influence of the moment of inertia of each layer is ignored.

### 2.2. Kinematics

**Figure 2:**
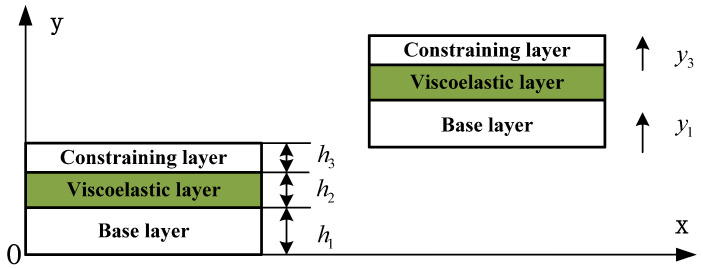
The geometric and deformation relationship of the EVES beam.

The geometric and deformation relationship of the EVES beam is shown in Figure 2.

According to the above assumptions, for the viscoelastic core layer, the transverse displacement of the mid-plane and the compressive strain can be respectively written as:

(1) $$y_{2}=\frac{1}{2}\left(y_{1}+y_{3}\right),\;\varepsilon_{2}=w_{1}-w_{3}$$

where $y_{2}$ and $\varepsilon_{2}$ are the transverse displacement and the compressive strain of the viscoelastic layer, respectively. $y_{1}$ and $y_{3}$ are the transverse displacement of the beam and the constraining layer, respectively.

### 2.3. Degrees of Freedom and Shape Functions

**Figure 3:**
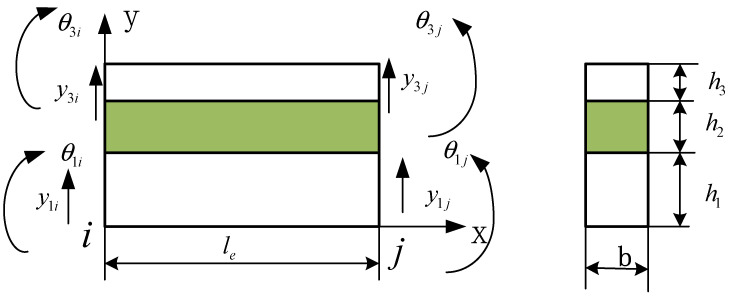
The element of the EVES beam.

The elementof the EVES beam is established, as shown in Figure 3. It is a sandwich composite element with two nodes. The length and width of the element are $l_{e}$ and b, respectively. The thickness of the base beam, viscoelastic layer, and the constraining layer of the element are $h_{1}$, $h_{2}$, and $h_{3}$, respectively. Each node of the element has four degrees of freedom (DOF), representing the transverse displacement and the rotation angle of the base beam layer and the constraining layer, respectively.

The node displacement vector of the element is given by

(2) $$\Delta^{e}={\left\{\begin{array}{cccccccc} y_{1i} & \theta_{1i} & y_{3i} & \theta_{3i} & y_{1j} & \theta_{1j} & y_{3j} & \theta_{3j} \end{array}\right\}}^{T}$$

In the element, the transverse displacement $y_{1}$ and $y_{3}$, the rotation angle $\theta_{1}$ and $\theta_{3}$ of the base beam layer and the constraining layer are expressed in the nodal displacements by FE shape functions

(3) $$y_{1}=N_{1}\Delta^{e},\;\theta_{1}=N_{2}\Delta^{e},\;y_{3}=N_{3}\Delta^{e},\;\theta_{3}=N_{4}\Delta^{e}$$

(4) $$N_{1}=\left[\begin{array}{cccccccc} 2{\left(\frac{x}{l_{e}}\right)}^{3}-3{\left(\frac{x}{l_{e}}\right)}^{2}+1 & \frac{x^{3}}{l_{e}{}^{2}}-2\left(\frac{x^{2}}{l_{e}}\right)+x & 0 & 0 & -2{\left(\frac{x}{l_{e}}\right)}^{3}+3{\left(\frac{x}{l_{e}}\right)}^{2} & \frac{x^{3}}{l_{e}{}^{2}}-\frac{x^{2}}{l_{e}} & 0 & 0 \end{array}\right]\;\;N_{2}=\left[\begin{array}{cccccccc} 6\left(\frac{x^{2}}{l_{e}{}^{3}}\right)-6\left(\frac{x}{l_{e}{}^{2}}\right) & 3{\left(\frac{x}{l_{e}}\right)}^{2}-4\left(\frac{x}{l_{e}}\right)+1 & 0 & 0 & -6\left(\frac{x^{2}}{l_{e}{}^{3}}\right)+6\left(\frac{x}{l_{e}{}^{2}}\right) & 3{\left(\frac{x}{l_{e}}\right)}^{2}-2\left(\frac{x}{l_{e}}\right) & 0 & 0 \end{array}\right]\;N_{3}=\left[\begin{array}{cccccccc} 0 & 0 & 2{\left(\frac{x}{l_{e}}\right)}^{3}-3{\left(\frac{x}{l_{e}}\right)}^{2}+1 & \frac{x^{3}}{l_{e}{}^{2}}-2\left(\frac{x^{2}}{l_{e}}\right)+x & 0 & 0 & -2{\left(\frac{x}{l_{e}}\right)}^{3}+3{\left(\frac{x}{l_{e}}\right)}^{2} & \frac{x^{3}}{l_{e}{}^{2}}-\frac{x^{2}}{l_{e}} \end{array}\right]\;\;N_{4}=\left[\begin{array}{cccccccc} 0 & 0 & 6\left(\frac{x^{2}}{l_{e}{}^{3}}\right)-6\left(\frac{x}{l_{e}{}^{2}}\right) & 3{\left(\frac{x}{l_{e}}\right)}^{2}-4\left(\frac{x}{l_{e}}\right)+1 & 0 & 0 & -6\left(\frac{x^{2}}{l_{e}{}^{3}}\right)+6\left(\frac{x}{l_{e}{}^{2}}\right) & 3{\left(\frac{x}{l_{e}}\right)}^{2}-2\left(\frac{x}{l_{e}}\right) \end{array}\right]$$

where the shape functions are given by substituting Equation (3) into Equation (1) gives

(5) $$y_{2}=N_{5}\Delta^{e},\;\varepsilon_{2}=N_{6}\Delta^{e}$$

where

(6) $$N_{5}=\frac{1}{2}\left(N_{1}+N_{3}\right),\;N_{6}=N_{1}-N_{3}$$

### 2.4. Energy Terms

#### 2.4.1. The Strain Energy

The potential energy from the bending of the base beam layer and the constraining layer are respectively written as

(7) $$U_{1}=\frac{1}{2}E_{1}I_{1}\int_{0}^{l_{e}}{\left(\frac{\partial^{2}y_{1}}{\partial x^{2}}\right)}^{2}dx=\frac{1}{2}\Delta^{e}{}^{T}K_{1}^{e}\Delta^{e},\;U_{3}=\frac{1}{2}E_{3}I_{3}\int_{0}^{l_{e}}{\left(\frac{\partial^{2}y_{3}}{\partial x^{2}}\right)}^{2}dx\;=\frac{1}{2}\Delta^{e}{}^{T}K_{3}^{e}\Delta^{e}$$

where $U_{1}$ and $U_{3}$, $E_{1}$ and $E_{3}$, $I_{1}$ and $I_{3}$ and the potential energy from bending, the elastic modulus, and the moment of inertia of the base beam layer and the constraining layer, respectively. $K_{1}^{e}$ and $K_{3}^{e}$ are the stiffness matrix of the base beam and the constraining layer, respectively. Applying the shape functions, they can be expressed in the form

(8) $$K_{1}^{e}=E_{1}I_{1}\int_{0}^{l_{e}}{\left[\frac{\partial^{2}N_{1}}{\partial x^{2}}\right]}^{T}\left[\frac{\partial^{2}N_{1}}{\partial x^{2}}\right]dx,\;K_{3}^{e}=E_{3}I_{3}\int_{0}^{l_{e}}{\left[\frac{\partial^{2}N_{3}}{\partial x^{2}}\right]}^{T}\left[\frac{\partial^{2}N_{3}}{\partial x^{2}}\right]dx$$

The viscoelastic layer is transversely compressed or stretched to dissipate energy. Therefore its potential energy from compression or stretching can be expressed as

(9) $$U_{2}=\frac{1}{2}\frac{E_{2}b}{h_{2}}\int_{0}^{l_{e}}{\left(y_{1}-y_{3}\right)}^{2}dx=\frac{1}{2}\Delta^{e}{}^{T}K_{2}^{e}\Delta^{e}$$

where $U_{2}$, $E_{2,}$ and $K_{2}^{e}$ are the potential energy, the elastic modulus, and the stiffness matrix of the viscoelastic layer, respectively. Applying the shape functions, and the $K_{2}^{e}$ can be obtained as

(10) $$K_{2}^{e}=\frac{E_{2}b}{h_{2}}\int_{0}^{l_{e}}\left(N_{6}{}^{T}N_{6}\right)dx=G_{v}\frac{2b\left(1+\nu_{2}\right)}{h_{2}}\int_{0}^{l_{e}}\left(N_{6}{}^{T}N_{6}\right)dx=G_{v}K_{vv}^{e}\;K_{vv}^{e}=\frac{2b\left(1+\nu_{2}\right)}{h_{2}}\int_{0}^{l_{e}}\left(N_{6}{}^{T}N_{6}\right)dx\;$$

where $G_{v}$, $v_{2}$, and $K_{vv}^{e}$ are the shear modulus, the Poisson’s ratio and the viscous stiffness matrix of the viscoelastic core layer.

Obviously, the total stiffness matrix of the element is the sum of the stiffness matrix of each layer. It can be written as

(11) $$K^{e}=\underset{K_{e}^{e}}{\underset{⏟}{K_{1}^{e}+K_{3}^{e}}}+K_{2}^{e}$$

where the sum of the first two terms on the right side of the equation is the elastic stiffness matrix $K_{e}^{e}$.

The total potential energy of the element is the sum of the potential energy of each layer. It can be written as

(12) $$U=U_{1}+U_{2}+U_{3}$$

#### 2.4.2. The Kinetic Energy

The kinetic energy from the transverse vibration of the element is given by

(13) $$T_{1}=\frac{1}{2}\rho_{1}A_{1}\int_{0}^{l_{e}}{\left(\frac{\partial y_{1}}{\partial t}\right)}^{2}dx=\frac{1}{2}{\dot{\Delta }}^{e}{}^{T}M_{1}^{e}{\dot{\Delta }}^{e}\;T_{2}=\frac{1}{2}\rho_{2}A_{2}\int_{0}^{l_{e}}{\left(\frac{\partial y_{2}}{\partial t}\right)}^{2}dx=\frac{1}{2}{\dot{\Delta }}^{e}{}^{T}M_{2}^{e}{\dot{\Delta }}^{e}\;T_{3}=\frac{1}{2}\rho_{3}A_{3}\int_{0}^{l_{e}}{\left(\frac{\partial y_{3}}{\partial t}\right)}^{2}dx=\frac{1}{2}{\dot{\Delta }}^{e}{}^{T}M_{3}^{e}{\dot{\Delta }}^{e}$$

where $T_{1}$, $T_{2}$, and $T_{3}$, $M_{1}^{e}$, $M_{2}^{e}$, and $M_{3}^{e}$ are the kinetic energy and mass matrices of the base beam layer, the viscoelastic layer, and the constraining layer, respectively. These mass matrices are written as

(14) $$M_{1}^{e}=\rho_{1}A_{1}\int_{0}^{l_{e}}N_{1}^{T}N_{1}dx,\;M_{2}^{e}=\rho_{2}A_{2}\int_{0}^{l_{e}}N_{5}{}^{T}N_{5}dx,\;M_{3}^{e}=\rho_{3}A_{3}\int_{0}^{l_{e}}N_{3}^{T}N_{3}dx$$

where $\rho_{1}$, $\rho_{2}$ and $\rho_{3}$, $A_{1}$, $A_{2},\;$ and $A_{3}$ are the density and the cross-sectional area of the base beam layer, the viscoelastic layer, and the constraining layer, respectively.

Obviously, the total mass matrix of the element is the sum of the mass matrix of each layer.

(15) $$M^{e}=M_{1}^{e}+M_{2}^{e}+M_{3}^{e}$$

The total kinetic energy of the element is the sum of the kinetic energy of each layer.

(16) $$T=T_{1}+T_{2}+T_{3}$$

### 2.5. Equation of Motion

The FE dynamic equation of viscoelastic composite structure can be expressed in Laplace domain as [15,16]

(17) $$\left(s^{2}M^{e}+K_{e}^{e}+s\tilde{G}\left(s\right)K_{vv}^{e}\right)X\left(s\right)=F^{e}\left(s\right)$$

where x(s) is the displacement vector, $F^{e}\left(s\right)$ is the excitation vector. $\;s\tilde{G}\left(s\right)=G^{*}\left(s\right)$ is the complex shear modulus of the viscoelastic material, which is frequency dependent.

The frequency-dependent characteristics of viscoelastic materials can be expressed by the Biot model

(18) $$s\tilde{G}\left(s\right)=G^{\infty }\left[1+\overset{N}{\underset{i=1}{\sum }}\frac{a_{i}s}{s+b_{i}}\right]$$

where $G^{\infty }$ is the steady-state value of the shear modulus of the viscoelastic material, *N* is the number of series items, $\left\{a_{i},b_{i}\right\}$ are positive constants.

Substituting the Biot model Equation (18) into Equation (17) and then introducing an auxiliary coordinate ${\hat{Z}}_{k}\left(s\right)=\frac{a_{k}}{s+b_{k}}X\left(s\right),\left(k=1,\;2,\;3\cdot \cdot \cdot N\right)$, the Formula (17) can be extended as

(19) $$\overset{-}{M}\overset{..}{q}+\overset{-}{D}\dot{q}+\overset{-}{K}q=\overset{-}{f}$$

where

(20) $$\overset{-}{M}=\left[\begin{array}{cccc} M^{e} & 0 & \cdots & 0\\ 0 & 0 & \cdots & 0\\ \vdots & \vdots & \ddots & \vdots \\ 0 & 0 & \cdots & 0 \end{array}\right],\;\overset{-}{D}=\left[\begin{array}{cccc} 0 & 0 & \cdots & 0\\ 0 & \frac{a_{1}}{b_{1}}\Lambda & \cdots & 0\\ \vdots & \vdots & \ddots & \vdots \\ 0 & 0 & \cdots & \frac{a_{N}}{b_{N}}\Lambda \end{array}\right]\;\overset{-}{K}=\left[\begin{array}{cccc} K_{e}^{e}+\tilde{K}\left(1+\overset{N}{\underset{K=1}{\sum }}a_{k}\right) & -a_{1}R & \cdots & -a_{N}R\\ -a_{1}R^{T} & a_{1}\Lambda & \cdots & 0\\ \vdots & \vdots & \ddots & \vdots \\ -a_{N}R^{T} & 0 & \cdots & a_{N}\Lambda \end{array}\right],\;q=\left\{\begin{array}{c} x\\ Z_{1}\\ \vdots \\ Z_{N} \end{array}\right\},\;\overset{-}{f}=\left\{\begin{array}{c} F^{e}\\ 0\\ \vdots \\ 0 \end{array}\right\}$$

where $\Lambda_{v}$ is a diagonal matrix, which is composed of the positive eigenvalues of the viscous stiffness matrix $K_{vv}^{e}$, and $R_{v}$ is a matrix with the corresponding orthogonal eigenvector as the column, $Z_{j}=R_{v}^{T}{\hat{Z}}_{j},\;\left(j=1,\;2,\;\cdots ,N\right)$.

Assembling all of the elements, the overall dynamic equation of the viscoelastic sandwich beam structure can be obtained as

(21) $$M\ddot{x}+D\dot{x}+Kx=F$$

where M, D and K  are the overall mass matrix, damping matrix, and stiffness matrix, respectively, F is the external force of the viscoelastic sandwich beam.

The numerical strategy used to extract the transverse vibration and damping characteristics was described in detail in reference [37].

## 3. Experiment

**Figure 4:**
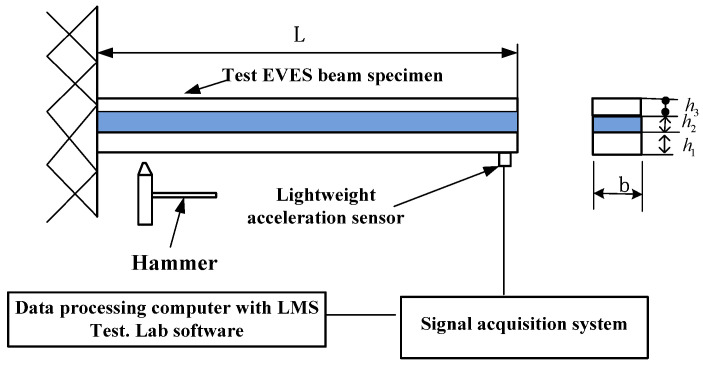
Experimental setup.

The experiment is designed to test the response of an EVES beam. Figure 4 shows the experimental setup. In the experiment, use a self-made lightweight, soft hammer with a rubber head to lightly hit the end of the EVES beam to excite vibration. A lightweight acceleration sensor is pasted at the free end to measure the vibration signal, which is collected and adjusted by the LMS SCADAS III 316 data acquisition system, and then analyzed by the LMS test. Lab vibration and noise test analysis system to obtain the natural frequencies and damping ratios.

The material and mechanical properties are shown in Table 1. The viscoelastic layer material is ZN-1, and its frequency-dependent Biot model parameters are given in reference [24]. The FE compression model is used to calculate the first three order natural frequencies and damping ratios of the EVES beam. A total of 30 finite elements are used for discreting it. The experimental and calculation results are listed in Table 2.

It can be seen from Table 2 that in the prediction of natural frequency, the calculation results are in suitable agreement with the experimental results. The prediction error is less than 4%. However, in the prediction of damping, the FE model shows deficiencies. It is only adequate for the first damping ratio, and for the second and third damping ratios, it shows a poor predictor of damping for the tested beam.

## 4. Numerical and Analytical Investigations

In order to study the compressive damping of EVES beams, Sisemore [35] carried out experiments on cantilever EVES beams with different thicknesses. They established an analytical model to study the transverse vibration. The thickness of the base beam layer of these beams is the same. The constraining layer and the viscoelastic layer have different thicknesses. The viscoelastic material is EAR-C1002. Table 3 shows the material parameters of the EVES beams, and Table 4 shows the thickness parameters.

Reference [38] shows that at the temperature of 23.9 °C, and the frequency range of f=0, ⋯, 2000 Hz, the storage modulus and loss factor of the complex shear modulus of the viscoelastic material layer (EAR-C1002) are, respectively,

(22) $${G_{v}}^{\prime }=\left(44.4-17.6/a\right)N/{\mathrm{mm}}^{2}$$

(23) $$\eta_{v}=1.643-0.6025z^{2}-\frac{0.2557\times 10^{-20}}{z^{16}}+\frac{0.1260\times 10^{-9}}{z^{8}}-\frac{0.1959\times 10^{-4}}{z^{4}}$$

where a=0.4+0.0003f, z=0.05+0.000475f.

The FE method presented in this paper is used to calculate the natural frequencies and damping ratios corresponding to the first two modes of the 9 beams listed in Table 2. The Biot model is used to describe the frequency-dependent characteristics of viscoelastic materials. Based on Equations (22) and (23), the Biot model parameters (Equation (18)) of EAR-C1002 can be fitted according to the method introduced in reference [24]. Table 5 lists these parameters.

Taking the experimental data in the reference [35] as the standard, the calculation results of the FE model presented in this paper are compared with the calculation results of the Sisemore model [35] and Mead-Markus model [16]. The last two models are both analytical models, in which the Sisemore modelisthe compression model, and the Mead-Markus model is the shear model. When calculating with the FE method in this paper, the EVES beams are discretized into 31 elements. The calculation results of the first two natural frequencies and damping ratios are presented in Table 6 and Table 7, respectively.

It can be seen from Table 6 that the accuracy of the compressional model is better than that of the Mead-Markus model in predicting the first two natural frequencies. Most of the prediction errors of the 9 beams are within 10%, the error range is 0.73–14%, and the average error is 7%. In comparison, the error of the Mead-Markus model is mostly larger than that of the compression model. The error range is 13–51%, and the average error is 28.5%. The error range of the Sisemore model is 0.1–11%, and the average error is 6%. The accuracy of the compressional model is close to the Sisemoremodel. This is because they are based on the same assumption of compressive energy dissipation.

According to Euler-Bernoulli beam theory, the first two natural frequencies of the base beam without additional damping layer and constraint layer is given by

(24) $$f_{n}=\frac{A_{n}}{2\pi L^{2}}\sqrt{\frac{EI}{\rho A}}$$

where $f_{n}\left(n=1,\;2\right)$ is the first two natural frequencies of the base beam (Hz), E is the Young’s modulus, I is the moment of inertia of section, ρ is the density, A is the cross-sectional area, $A_{n}=\left\{3.52,\;22.4\right\}$ is modal factor. Substituting the material parameters of the base beam in Table 1 into Equation (24), the first two natural frequencies of the base beam are 53 and 333 Hz. With the addition of the viscoelastic layer and the constraining layer, the stiffness and mass increase accordingly. It can be seen from Equation (24) that the increase in additional stiffness will cause the natural frequency of the system to increase, but the increase in additional mass will lead to the decrease in the natural frequency. It can be seen from Table 1 that the density of the viscoelastic layer is about 50% of the base beam. However, the stiffness of the viscoelastic layer is only 0.01% of it. Obviously, the effect of the additional mass on the natural frequency far exceeds the additional stiffness. Therefore, after adding a viscoelastic layer, the natural frequency of the structure will decrease. It can be seen from Table 4 that the natural frequency values obtained by the experiment are all smaller than the theoretical value of the natural frequency of the base beam, which is in line with the above analysis. The predicted value of the natural frequency of the FE compression model is very close to the experimental results. On the contrary, the natural frequencies of most sandwich beams predicted by the shear model are higher than those of the base beam, and the error is larger than that of the compression model. Therefore, it is obvious that under the geometric conditions, the shear model will overestimate the natural frequency of the EVES beams, while the compression model has higher accuracy.

Table 7 presents a comparison of the predicted and experimental damping ratios of the nine EVES beams. The results indicate that all three models are not accurate enough to predict the damping ratio. However, some laws can still be seen from it. In the prediction of the damping ratio corresponding to the first-order natural frequency of the beams, the compression damping model only predicts beams 3, 6, and 9 relatively accurately. The common point of the three beams is that their constraint layers have the maximum thickness. This shows that when the stiffness of the constrained layer increases, the prediction accuracy of the compression damping model will improve. This is because when the stiffness of the constraint layer increases, it helps to make the viscoelastic layer produce transverse tension and compression deformation, which is closer to the assumption of the compression damping model. When the constraining layer is relatively thin, its stiffness is small, and at this time, it will produce bending deformation under the action of the viscoelastic layer, which is contrary to the assumption of the compression damping model. Therefore, in beams 3, 6, and 9, compression damping accounts for the main proportion. In addition, it can be seen that in these three beams, when the viscoelastic layer is thicker, the compression damping is more obvious, and the prediction accuracy of the FE compression model for the damping ratio is higher. Similarly, in beams 1, 4, and 7, the thickness of the constrained layer is the smallest, and the prediction accuracy of the FE compression model is the worst. In addition, the average prediction error of theMead-Markus model for the first-order damping ratio exceeds 130%. Obviously, the shear model overestimates the first-order damping ratio of the EVES beams. This overestimation may have a bad influence on the design and optimization of the EVES beam structures.

In the prediction of the second-order damping ratio, the compression models for all beams are distorted. However, in comparison, the Mead-Markus model is more adequate. This shows that in the second-order mode, the shear damping accounts for the main proportion, and in this case, the compression model is not applicable. This can be explained by the vibration mode of the cantilever beam. In the second-order vibration mode, the constraining layer and the foundation beam are more likely to produce relative axial displacement. In this case, the shear deformation of the viscoelastic layer dissipates more vibration energy, which means that the shear damping is dominant. This also shows that the vibration mode is also a factor that determines the damping mechanism of the EVES beam. This also shows the complexity of the damping mode of the viscoelastic sandwich beam, which is related not only to the thickness of each layer of the structure but also to the vibration mode.

## 5. Conclusions

A FE compressional model of EVES beams is established to study its transverse vibration and damping characteristics. The FE is a three-layer two-node beam element with four degrees of freedom at each node. It is considered that the viscoelastic material is compressible, and the damping of the EVES beam is only caused by the transverse compression/tension of the viscoelastic layer.Biot model is used to describe the frequency-dependent characteristics of viscoelastic materials. Nine numerical examples are introduced to compare the calculation results of the FE model in this paper with those of a compressional analytical model and a shear analytical model. Finally, an EVES beam is experimentally studied. Some conclusions are obtained:

1. The transverse compressional vibration is a broadband existence in EVES beams;
2. The FE compressional model presented inthis paper is in suitable agreement with the analytical model. It is a relatively accurate and simple method for predicting the natural frequencies of EVES beams. However, it is quite poor for damping predictions because it ignores shear damping;
3. The geometry and the vibration mode determine the damping mechanisms. The shear model and the compressional model have different applicable conditions, which are related to the thickness of each layer and the vibration mode of the structure. In general, the shear energy dissipation assumption is applicable to EVES thin-walled beams, and the compression energy dissipation assumption is applicable to EVES beams with relatively thick constraining layer and base beams;
4. When the base beam and the constrained layer are relatively thick, and the viscoelastic layer is relatively thin, neither the current shear model nor the compression model can accurately predict the damping. A model including shear damping and compressional damping should greatly improve the accuracy of damping prediction, which needs further research.

The EVES structure studied in this paper is in a simple environment. In fact, they sometimes work in a state of motion or a special environment. When they are moving in a straight line or rotating, their dynamic characteristics need further research. In addition, when the structure is in strong airflow, flutter may occur, and buckling may occur in an aero-thermal environment. These special behaviors also need to be further studied. The work proposedin this paper can be used in these studies.

## Figures and Tables

**Table 1:** Material parameters of experimental specimen.

Material Properties	Constraining Layer	Base Beam	Viscoelastic Layer (ZN-1)
Elastic modulus (GPa)	699	699	Biotmodel [24]
Density (kg/m^3^)	2700	2700	1010
Poisson’s ratio	0.3	0.3	0.3
Thickness (mm)	4.91	4.85	4.91
Length (mm)	290	290	290
Width (mm)	25	25	25

**Table 2:** Comparison of experimental results and FE calculations.

Order	Experimental Result		FE Model This Paper			
	Natural Frequency (Hz)	Damping Ratio	Natural Frequency (Hz)	Error (%)	Damping Ratio	Error (%)
1	52.5	0.1350	51.6	1.7	0.1265	6.3
2	315.5	0.0846	279.8	1.1	0.0124	85.3
3	842.0	0.0546	747.5	3.36	0.0009	98.4

**Table 3:** Structure and material parameters of cantilever EVESbeams.

Material Properties	Constraining Layer	Base Beam	Viscoelastic Material Layer(EAR-C1002)
Elastic modulus (GPa)	71	71	Frequency dependent
Density (kg/m^3^)	2710	2710	1280
Poisson’s ratio	0.3	0.3	0.3
Length (mm)	314	314	314
Width (mm)	25.4	25.4	25.4

**Table 4:** Thickness of each layer of cantilever EVES beams.

Number	Thickness of Base Beam (mm)	Thickness of the Viscoelastic Layer (mm)	Thickness of the Constraining Layer (mm)
1	6.350	0.381	1.588
2	6.350	0.381	3.175
3	6.350	0.381	6.350
4	6.350	3.048	1.588
5	6.350	3.048	3.175
6	6.350	3.048	6.350
7	6.350	6.350	1.588
8	6.350	6.350	3.175
9	6.350	6.350	6.350

**Table 5:** Fitted parameters of the Biotmodel for EAR-C1002 at 23.9 °C.

	k=1	k=2	k=3
*G^∞^*	4 × 10^5^		
*a_k_*	8.2244	1.1116 × 10^3^	4.8334 × 10^2^
*b_k_*	2.2936 × 10^5^	1.7267 × 10^6^	5.9245 × 10^6^

**Table 6:** Natural frequencies of the first two modes for cantilever EVES beams: comparison of experimental and calculated results.

Beam	Experiment [35]	Sisemore Model [35]		Mead-Markus Model [16]		Compressional Model	
	Frequency (Hz)	Frequency (Hz)	Error (%)	Frequency (Hz)	Error (%)	Frequency (Hz)	Error (%)
First natural frequency							
1	47.3	47.3	0.1	55.7	18	47.8	1.0
2	44.5	45.2	1.5	56.6	27	43.8	1.6
3	44.2	48.5	9.7	66.9	51	48.6	9.9
4	43.0	44.0	2.4	48.4	13	44.5	3.4
5	40.9	42.3	3.6	47.6	17	41.2	0.73
6	40.7	44.1	8.5	55.4	36	45.6	12
7	39.6	40.7	2.8	45.0	14	41.2	4.0
8	38.1	39.5	3.6	44.4	17	38.5	1.0
9	37.8	41.0	8.5	52	38	41.7	10
Second natural frequency							
1	329	297	9.8	376	14	299	9.0
2	319	284	11	423	33	274	14
3	350	313	11	528	51	301	14
4	293	276	6.0	357	22	278	5.1
5	284	266	6.2	364	28	257	9.5
6	305	293	4.0	419	37	280	8.2
7	270	255	5.3	344	28	258	4.4
8	262	249	5.0	344	32	241	8.0
9	286	276	3.6	388	36	259	9.4

**Table 7:** Damping ratios of the first two modes for cantilever EVES beams: comparison of experimental and calculated results.

Beam	Experiment [24]	Sisemore Model [24]		Mead-Markus Model [11]		Compressional Model	
	Damping Ratio	Damping Ratio	Error (%)	Damping Ratio	Error (%)	Damping Ratio	Error (%)
Damping ratio corresponding to the first natural frequency							
1	0.0223	0.001	2200	0.0462	110	0.0146	35
2	0.0267	0.0015	1700	0.0768	190	0.0197	26
3	0.0215	0.0151	42	0.0914	330	0.0190	12
4	0.0214	0.0002	11000	0.0576	170	0.0288	35
5	0.0226	0.0023	880	0.0663	190	0.0326	44
6	0.0226	0.0203	11	0.0663	180	0.0271	20
7	0.0229	0.0003	7500	0.0663	190	0.0308	34
8	0.0224	0.0028	700	0.0702	210	0.0325	45
9	0.0231	0.0223	4	0.0596	160	0.0242	5
Damping ratio corresponding to the second natural frequency							
1	0.0513	9 × 10^−5^	57000	0.0392	24	0.0551	7
2	0.0826	0.0011	74000	0.0654	21	0.0765	7
3	0.1084	0.0073	1400	0.0809	25	0.0801	26
4	0.0914	0.0002	46000	0.0809	7	0.0879	4
5	0.1055	0.0015	6900	0.0980	18	0.1303	23
6	0.1027	0.0074	1300	0.1243	24	0.1474	43
7	0.0977	0.0005	19000	0.1274	23	0.1287	31
8	0.0967	0.0018	5300	0.1205	77	0.1218	26
9	0.1070	0.0072	1400	0.1707	12	0.1350	26

## Data Availability

Data sharing not applicable.
